# Hydroxymethylation alterations in progenitor-like cell types of pediatric central nervous system tumors are associated with cell type-specific transcriptional changes

**DOI:** 10.21203/rs.3.rs-2517758/v1

**Published:** 2023-02-28

**Authors:** Min Kyung Lee, Nasim Azizgolshani, Ze Zhang, Laurent Perreard, Fred W. Kolling, Lananh N. Nguyen, George J. Zanazzi, Lucas A. Salas, Brock C. Christensen

**Affiliations:** 1 Department of Epidemiology, Geisel School of Medicine at Dartmouth, Lebanon, NH, USA; 2 Department of Cardiothoracic Surgery, Columbia University Medical Center, New York, NY, USA; 3 Dartmouth Cancer Center, Geisel School of Medicine at Dartmouth, Lebanon, NH, USA; 4 Department of Laboratory Medicine and Pathobiology, University of Toronto, Toronto, ON, Canada; 5 Department of Pathology and Laboratory Medicine, Geisel School of Medicine at Dartmouth, Lebanon, NH, USA; 6 Department of Molecular and Systems Biology, Geisel School of Medicine at Dartmouth, Lebanon, NH, USA; 7 Department of Community and Family Medicine, Geisel School of Medicine at Dartmouth, Lebanon, NH, USA

## Abstract

Although intratumoral heterogeneity has been established in pediatric central nervous system tumors, epigenomic alterations at the cell type level have largely remained unresolved. To identify cell type-specific alterations to cytosine modifications in pediatric central nervous system tumors we utilized a multi-omic approach that integrated bulk DNA cytosine modification data (methylation and hydroxymethylation) with both bulk and single-cell RNA-sequencing data. We demonstrate a large reduction in the scope of significantly differentially modified cytosines in tumors when accounting for tumor cell type composition. In the progenitor-like cell types of tumors, we identified a preponderance differential CpG hydroxymethylation rather than methylation. Genes with differential hydroxymethylation, like *HDAC4* and *IGF1R*, were associated with cell type-specific changes in gene expression in tumors. Our results highlight the importance of epigenomic alterations in the progenitor-like cell types and its role in cell type-specific transcriptional regulation in pediatric CNS tumors.

## Introduction

Central nervous system (CNS) tumors are the leading cause of cancer death in the pediatric population^1^. While major progress has been made in reducing the mortality in pediatric cancers in the past few decades, the magnitude of reduction in the mortality rate of CNS tumors have not been as substantial^2^. Even among patients who survive childhood cancers, those who have survived CNS tumors have the highest cumulative burden of disease post-survival^3^. Craniospinal radiation and neurotoxic therapy are major risk factors for the future burden on quality of life with late effects including neurocognitive impairments such as academic and memory decline, and adverse health outcomes like abnormal hearing and growth hormone deficiency^4–9^. Efforts to address discrepancies in the reduction of mortality rates and extensive chronic health burdens later in life have been made with the recent advances in technology that have allowed for better insight into the molecular characterization of pediatric CNS tumors^10–22^. Molecular biomarkers are progressively being incorporated into the diagnosis and management of certain pediatric CNS tumor types^23^.

One method to supplementally diagnose and subtype CNS tumors is DNA methylation^24^. Capper et al. developed a classification method to address previous issues in inter-observer variability for histopathological diagnosis of many CNS tumors^24^. Since the development of this method, DNA methylation classification is now used regularly for certain pediatric CNS tumor types, like ependymomas, to understand the prognosis and manage treatment decisions^13,14^. This method utilizes bisulfite-treated DNA, which does not distinguish between 5-methylcytosine and 5-hydroxymethylcytosine, although it has been indicated only 5-methylcytosine signal from oxidative bisulfite-treated DNA alters the classification from this method^25,26^. Moreover, while advancements have improved management strategies for some tumor types, many other pediatric CNS tumor types remain underexplored.

DNA methylation is one of the most well-studied epigenomic marks, primarily known for its role in regulating gene expression. DNA methylation occurs when a methyl group is added to the 5-carbon position of a cytosine in the context of a Cytosine-phosphate-Guanine (CpG) dinucleotides by DNA methyltransferases (DNMTs)^27–32^. Methylation of CpG island promoters is associated with repression of gene expression while methylation of gene bodies is associated with activation of gene expression^33–35^. 5-methylcytosine (5-mC) many times co-exist with H3K9me3 marks and do not overlap with H3K4me3 marks and H2A.Z^34,36,37^. In addition, DNA methylation marks function as genome stabilizers by silencing transposable elements^34,38^. The main ways DNA methylation is altered in cancer include genome-wide hypomethylation in repetitive elements like retrotransposable elements^39,40^, hypermethylation of promoters^40–43^, and propensity for cytosines in CpG contexts to be mutated^44–47^.

Cytosines can also remain in a hydroxymethylated state (5-hydroxymethylcytosine, 5-hmC). 5-hmC is formed when 5-mC is actively being demethylated by ten-eleven translocation (TET) enzymes^48–50^. TET enzymes add a hydroxyl group onto the methyl group to become 5-hydroxymethylcytosine, then add the hydroxyl group again to become 5-formylcytosine, then again to become 5-carboxylcytosine, which is excised to become unmethylated^48–51^. While 5-hmC is an intermediate, it has been shown to have functional roles and be stable in the genome. Like 5-mC, 5-hmC has been associated with regulating transcription. It is enriched in gene bodies of active genes and in transcription start sites in which promoters are marked with H3K27me3 and H3K4me4^52,53^. 5-hmC has also been shown to play roles in maintaining pluripotency and tumorigenesis^52,54^. While generally 5-hmC levels are relatively much lower than 5-mC levels, higher levels of 5-hmC are found in the brain tissue compared to other tissue and in embryonal stem cells developmentally programmed neuronal cells^52,55–61^. Although progress has been made since the discovery of TET enzymes producing 5-hmC^49–51^, more investigation is needed to understand the functional roles of 5-hmC. While alterations in hydroxymethylation patterns have not been as well examined, studies have indicated decreased hydroxymethylation across the genome in a variety of tumor types including adult and pediatric CNS tumors^26,54,62–70^, and mutations in hydroxymethylation-associated genes such as *IDH1/2* and *TET1/2/3* have been associated with certain tumor types like gliomas and acute myeloid leukemia^62,71–74^.

Numerous studies have established that brain tumors display intratumoral cellular heterogeneity^17,19,20,75–85^. While it is known that both DNA methylation and hydroxymethylation patterns are tissue type and cell type dependent^52,53,86–90^, limited research has addressed cell type-specific DNA cytosine modification alterations in these tumors. This gap exists largely due to the high cost and limitations in technologies to profile cytosine modifications at the cell type-specific scale^91^. While the importance of cell type composition effects in epigenome-wide association studies has been well documented^92–96^, single-cell methylation profiling strategies^97–100^ are slowly developing in comparison to more accessible and commercially available genome profiling technologies focused on gene expression or chromatin accessibility. To address these shortcomings, computational methods have been developed to deconvolute cell type composition using DNA methylation for certain tissue types^91,101–109^. While these methods have greatly improved our understanding of the cell type composition effects on many epigenome-wide association studies, they have not been utilized in investigating cell type composition effects on brain tumors due to some limited applicability in brain tissue.

In this study, we use a multi-omic approach to study cell type-level epigenomic alterations in pediatric CNS tumors to maximize the applicability of currently available methods. By integrating single nuclei RNA-seq and cytosine modification data, we provide a more complete picture of the cytosine modification alterations associated with pediatric CNS types and cytosine modifications that are associated with changes in transcription at the cell type level in pediatric CNS tumors.

## Results

To assess the potential normal tissue margin in our tissues that may confound downstream analyses, we first determined the tumor purity of our pediatric CNS tumor samples that were used to measure DNA cytosine modifications. Tumor purity in our samples varied but did not significantly differ based on tumor type or grade (**Supplementary Figure 1**).

### Genomic burden altered cytosine modifications

To determine the global epigenomic burden of altered cytosine modifications in pediatric CNS tumors compared to non-tumor pediatric brain tissue, we compared median beta values for both 5-hmC and 5-mC across samples at each CpG and determined the methylation dysregulation index (MDI). MDI is a summary measure of the epigenome-wide alteration of tumors compared to non-tumor tissue^110^. Tumor tissues displayed a decrease in 5-hmC and a slight increase in 5-mC compared to non-tumor tissue (**Figure 1A**). The 5-hmC MDI values were not significantly different by tumor type or by tumor grade (**Figure 1B**), whereas 5-mC MDI values varied by tumor type. Embryonal tumors had the greatest extent of epigenome-wide alteration burden compared to non-tumor tissue, astrocytomas had the lowest burden of 5-mC MDI compared to non-tumor tissue, and we observed increasing 5-mC MDI with increasing tumor grade. 5-hmC MDI and 5-mC MDI were positively correlated (R = 0.44, p-value = 0.013, **Figure 1C**). We repeated our analysis after removing one astrocytoma sample with an outlier 5-hmC MDI value and observed consistent results (**Supplementary Figure 2**). We tested and confirmed that the burden of observed epigenomic alterations was not due to differences in tumor purity, (**Supplementary Figure 3, Supplementary Table 2A**). However, we did observe significant differences in 5-mC MDI by tumor grade (**Supplementary Table 2B**). While 5-hmC is prevalent at only 6% of 5-mC, the level of dysregulation of the hydroxymethylome is comparable to the level of dysregulation of the methylome with 5-hmC MDI being 49% of 5-mC MDI (**Table 2**). Our results suggest that while 5-hmC may not be as prevalent, epigenome-wide alterations of 5-hmC in tumors are occurring at comparable levels to altered 5-mC.

### Cell type composition influences bulk-omics comparisons between pediatric CNS tumors and non-tumor pediatric brain tissue

We utilized our single nuclei RNA-seq data to identify the cell type composition of pediatric CNS tumor tissue and non-tumor pediatric brain tissue. Based on the cell type proportion distributions for all of our samples, we identified neuronal-like cells (NEU), neural stem cells (NSC), oligodendrocyte precursor cells (OPC), radial glial cells (RGC), and unipolar brush cells (UBC) as having the most variance (**Supplementary Figure 4A**). For each tumor type we compared proportions of cell types with non-tumor pediatric brain tissue. Supporting our principal component analysis, the cell types with the greatest differences were NEU, NSC, OPC, RGC, and UBC (**Supplementary Figure 4B**).

We conducted an epigenome-wide association study to determine the differential hydroxymethylated and methylated CpGs associated with each tumor type compared to non-tumor pediatric brain tissue. To reduce potential confounding by cell type composition, we incorporated cell type proportions as covariates in a stepwise manner to each series of linear models. Importantly, as the number of cell type proportion covariates included in the models increased, the scope of differentially hydroxymethylated and differentially methylated CpGs associated with each tumor type decreased (**Figure 2A – 2D, Supplementary Figure 5 – 8**). In addition, across our models in different tumor types, the extent of differentially hydroxymethylated CpGs (dhmCpGs) was far greater than that of differentially methylated CpGs (dmCpGs). When all five cell types (NEU, NSC, OPC, RGC, and UBC) were incorporated into the model, we observed low number of dmCpGs associated with each tumor type. Embryonal tumors had the greatest number of dhmCpGs, and the 83.1% were specific to the embryonal tumors (**Figure 2E**). In the model with all five cell types included, 87 dhmCpGs were associated with astrocytoma, 850 dhmCpGs were associated with embryonal tumors, 31 dhmCpGs were associated with ependymoma, and 126 dhmCpGs were associated with glioneuronal/neuronal tumors. We identified 90 dhmCpGs (10.4%) that were shared across two or three of the tumor types and 28 dhmCpGs (3.2%) that were shared across all tumor types (**Figure 2E**). Our results suggest that epigenome-wide association studies comparing bulk pediatric CNS tumor tissue to non-tumor pediatric tissue are considerably influenced by the cell type composition. Moreover, it was quite unexpected that the observed differences were almost solely in hydroxymethylation and not in methylation.

We then compared transcriptome data from bulk RNA-seq in each of the tumor types with non-tumor pediatric brain tissue. The differential expression testing model included the same covariates (sex, age at diagnosis, and tumor purity) and the same five cell type proportions used for the EWAS analysis. Including proportions of major cell types of interest led to differences in an average of around 702 genes (range: 536 – 892) detected as significantly differentially expressed. In astrocytoma and glioneuronal/neuronal tumors, the adjusted model identified more genes that were significantly differentially expressed. In embryonal tumors and ependymomas, the adjusted model identified fewer genes that were significantly differentially expressed. Some key tumor progression-associated genes like *PTEN* in astrocytoma and in embryonal tumors, *MYCN* in ependymoma, and *BRCA2* in glioneuronal/neuronal tumors would not otherwise have been identified as significantly differentially expressed in the tumors had the cell type proportions not been adjusted for.

Across all tumor types, the majority of differentially expressed genes were increased in expression compared to the non-tumor pediatric brain tissue (**Supplementary Figure 9A, 10 - 13**). Almost half (43%, 3020 genes) of all genes with increased expression were shared across all tumor types (**Supplementary Figure 9B**). Among the genes with shared increases in expression in tumors were *IRX5, MYOSLID, CWH43, ITGA2*, and *H0XA3*. Genes with increased expression across all tumor types were associated with biological oxidations and keratinization among other pathways (**Supplementary Figure 9D**). There were 253 genes (13.6%) that had decreased expression shared across tumor types (**Supplementary Figure 9C**), including *NPTXR, SCG2, B4GAT1*, and *ATRN*. Genes that were decreased in expression across all tumor types were associated with the insulin receptor signaling and ion channel transport among other pathways (**Supplementary Figure 9E**).

To identify potentially important gene regulation by differential hydroxymethylation we compared changes in hydroxymethylation in dhmCpGs from the five-cell type-adjusted model with gene expression in each tumor type. Generally, genes with decreased hydroxymethylation levels had increased gene expression across tumor types compared to non-tumor pediatric brain tissue (**Figure 3**). Only one dhmCpGs associated with ependymoma had significant decreased expression. The dhmCpGs with differential expression did not generally favor promoters or gene body regions (**Figure 3, Supplementary Table 3**). Only embryonal tumors displayed slightly varying associations. While many of the dhmCpGs associated with embryonal tumors followed similar patterns of decreased 5-hmC levels and increased gene expression, there were some CpGs with decreased 5-hmC and decreased gene expression, as well as CpGs with increased 5-hmC with increased or decreased gene expression levels. Embryonal tumor associated dhmCpGs with significantly increased gene expression were less likely to be in promoter regions compared to dhmCpGs with significantly decreased gene expression (OR (95%CI) = 0.23 (0.064 – 0.78), p-value = 0.01). On the contrary, embryonal tumor associated dhmCpGs with significant increased expression were marginally more likely to be in gene body regions (OR (95%CI) = 2.81 (0.84 – 10.34), p-value = 0.06). We could not test for associations between promoter or gene body regions for other tumor types due to the limited number of dhmCpGs.

Interestingly, there were two CpGs with decreased 5-hmC levels and increased gene expression in astrocytoma, ependymoma, and glioneuronal/neuronal tumors: cg18280362 located in the promoter region of *CWH43* and cg08278401 located in the promoter region of *LRRC72*. In addition, we investigated the association between changes in 5-mC methylation and gene expression in the embryonal tumors where there were 24 dmCpGs associated with significant changes in gene expression (**Supplementary Figure 14**). While we could not conduct statistical tests to test for an enrichment of promoter/gene body regions for shared dhmCpGs with increased gene expression, there were 18 dhmCpGs with increased gene expression in non-promoter regions and 3 dhmCpGs with increased gene expression in promoter regions. Moreover, there were 9 dhmCpGs with increased gene expression not in gene body regions and 12 dhmCpGs in gene body regions (**Supplementary Table 3**). Our results indicate that hydroxymethylation may be associated with changes in gene expression for certain genes in pediatric CNS tumors.

### Molecular alterations in pediatric CNS tumors occur in a cell type-specific and tumor type-specific manner

One of the major questions that remains unanswered in many epigenome-wide association studies is whether altered cytosine modification can be ascribed to a specific cell type. With data from single nuclei RNA-seq for these pediatric CNS tumors and non-tumor pediatric brain tissues, we sought to identify epigenomic alterations at a cell type-specific level. To reduce the number of covariates in our analysis we focused on neuronal-like and progenitor-like cell types (**Supplementary Table 4**). The progenitor-like cells were an aggregation of neural stem cells, radial glial cells, oligodendrocyte precursor cells, and unipolar brush cells. We used an approach developed by Zheng et al^103^ called CellDMC to identify cell-type-specific differentially hydroxymethylated and methylated CpGs. Using CellDMC we identified abundant dhmCpGs for each cell type and tumor type, far greater than the scope of CpGs identified with bulk tissue EWAS (**Figure 4A, Supplementary Figures 15 – 18, Supplementary Table 5**). While there were a relatively lower number of dmCpGs compared to the dhmCpGs, there were some dmCpGs detected in the cell type-specific model (**Figure 4B**). Majority of the cell type-specific dhmCpGs were tumor-type-specific (**Figure 4C – 4D, Supplementary Figure 19**). However, 128 dhmCpGs were observed in the neuronal-like cell types and 534 dhmCpGs were observed in the progenitor-like cell types across all four tumor types. While some neuronal-like cell-specific dhmCpGs were acting on the same genes as the progenitor-like cell-specific dhmCpGs, genes that had decreased 5-hmC in the progenitor-like cells were exclusive (**Supplementary Figure 20**).

We then assessed the genomic context of cell type-specific dhmCpGs and tested for enrichment to various genomic contexts stratified by the direction of differential hydroxymethylation. Interestingly, both increased and decreased dhmCpGs in neuronal-like and progenitor-like cell types of astrocytoma and glioneuronal/neuronal tumors were enriched in similar contexts at Dnase hypersensitive sites (DHS), 1^st^ exons, promoter regions (TSS200, TSS1500), and 5’ UTR regions (**Figure 5**). dhmCpGs in ependymoma were dependent on the cell type in which it was occurring. Ependymoma associated dhmCpGs in the neuronal-like cells and CpGs with increased 5-hmC in progenitor-like cells were enriched in similar regions as the astrocytoma and glioneuronal/neuronal tumors. On the contrary, ependymoma associated CpGs with decreased 5-hmC in the progenitor-like cells were enriched in transcription factor binding sites (TFBS), 3’ UTR, gene body, and exon regions. The dhmCpGs, especially for those occurring in the progenitor-like cell types, in embryonal tumors were enriched in distinct genomic contexts compared to the other tumor types. Progenitor-like cell type-specific dhmCpGs were enriched in the transcription factor binding sites, 3’ UTR, gene body, exons, and enhancers.

Our findings indicate that most of the hydroxymethylation alterations occur in the progenitor-like cell types and are tumor-type-specific.

### Cell type-specific gene expression changes associated with changes in hydroxymethylation

We next evaluated cell-specific gene expression changes for genes with cell-type-specific changes in hydroxymethylation. We calculated gene expression scores for genes associated with CpGs with differentially hydroxymethylated CpGs in the neuronal-like cells and progenitor-like cells for each granular cell types incorporated in our analysis for each tumor type (**Supplementary Figures 21 – 25**). Interestingly, for all tumor types, the expression scores for genes associated with CpGs with increased or decreased hydroxymethylation were increased in the oligodendrocyte precursor cells (OPCs) of the tumors compared to non-tumor pediatric brain tissue (**Figure 6A**). Only the OPCs in embryonal tumors did not show a statistically significant increase in the expression of genes with increased 5-hmC in the progenitor-like cells. On the contrary, gene expression levels for each of the gene sets with cell type-specific alterations in 5-hmC were decreased in each of the cell types for all tumors compared to the non-tumor pediatric brain tissue.

*HDAC4*, established as associated with cancer progression and poor prognosis in a variety of tumor types^111–119^, was one gene with cell type-specific dhmCpGs across all four tumor types. Interestingly, the majority of the CpGs with decreased 5-hmC were associated with progenitor-like cell types, while the majority of the CpGs with increased 5-hmC were associated with the neuronal-like cell types in the tumor tissue (**Figure 6B**). More than 50% of the dhmCpGs in *HDAC4* for each tumor type were in the gene body (**Table 3**). There were few dhmCpGs in the 5’ UTR, TSS200, and DNase hypersensitive sites (DHS). The neuronal-like cell types had lower expression of *HDAC4* across all tumor types compared to the non-tumor tissue (**Figure 6D**). On the contrary, the progenitor-like cell types had higher levels of *HDAC4* expression.

*IGF1R* had dhmCpGs across all tumor types and is associated with tumorigenesis, therapy resistance, and poor survival in different cancer types, including in some pediatric CNS tumor types^120– 130^. Most of the dhmCpGs with decreased 5-hmC were associated with the progenitor-like cell types in the tumor tissue while only a couple dhmCpGs were in the neuronal-like cell types of the tumor tissue (**Figure 6C**). Like *HDAC4*, the dhmCpGs in *IGF1R* were mostly located in the gene body and DNase hypersensitive sites, with a few scattered in the enhancer and 3’ UTR regions (**Table 4**). Consistent with the lack of changes in hydroxymethylation in the neuronal-like cell types of the tumors, gene expression levels of *IGF1R* did not differ between tumors and the non-tumor tissue among neuronal-like cell types (**Figure 6D**). However, following the decreases in hydroxymethylation, *IGF1R* gene expression levels were higher in the progenitor-like cell types, particularly the OPCs, in the tumors than in the progenitor-like cell types of non-tumor tissue. EWAS results from bulk tumor tissue identified only one or two CpGs in *HDAC4* and *IGF1R* as differentially hydroxymethylated in either cell type-adjusted or unadjusted model (**Table 4**).

Our results suggest potentially critical roles of hydroxymethylation of CpGs located within the gene body regions in regulating the gene expression of critical cancer genes, like *HDAC4* and *IGF1R*.

## Discussion

In this study, we investigated the cell type-specific cytosine modification alterations in pediatric central nervous system tumors with a multi-omic approach. We described the cell type composition effects that occur in epigenome-wide association studies using bulk pediatric central nervous system tumors and non-tumor pediatric brain tissue. We identified that there were more differentially hydroxymethylated CpGs associated with each tumor type, particularly in the progenitor-like cell types, rather than differentially methylated CpGs. Lastly, we show that the cell type-specific changes in hydroxymethylation are associated with cell type-specific gene expression changes in pediatric central nervous system tumors.

Based on methods to classify tumor subtypes and the predominant focus on DNA methylation, it was unexpected that there were very few differentially methylated CpGs associated with each tumor type. One possible explanation for this phenomenon may be that as these are pediatric tissues, there is still ongoing development with which 5-hmC is associated. As our results suggest the epigenome-wide alterations of 5-hmC in these tumors, it may be critical to distinguish between 5-mC and 5-hmC to better understand the molecular underpinnings of these pediatric CNS tumors. Furthermore, it may be beneficial to incorporate 5-hmC into cytosine modification-based classification methods to improve performance.

Pediatric tumors are known not to have substantial genetic alterations. Our results suggest that pediatric CNS tumors may be characterized by non-mutational epigenomic reprogramming more so than genomic aberrations^131,132^. We identified a substantial number of differentially hydroxymethylated CpGs associated with progenitor-like cell types of each tumor type. Additionally, even among the shared differentially hydroxymethylated CpGs in the progenitor-like cell types, numerous differentially hydroxymethylated CpGs were located within different genes that regulate epigenetic patterns, such as *DNMT3A, HDAC4, MLLT3*, and *KAT2B*. Furthermore, pediatric brain cancers have been shown to contain somatic mutations in epigenetic regulator genes such as *H3F3A, KDM6A*, and MLL5^133–135^. Considering the dysregulation of the epigenome may be important when developing new therapeutic strategies for these tumors.

While much more investigation has been conducted into how DNA methylation regulates gene expression, less is known about how DNA hydroxymethylation can also be associated with changes in gene expression. We identified relationships between cell type-specific hydroxymethylation patterns and cell type-specific gene expression in our pediatric CNS tumors. Our findings indicate that hydroxymethylation changes in the gene body regions can alter gene expression. Previous studies have found positive associations between DNA methylation in gene body regions and gene expression changes^33,44^. However, many genome-wide DNA methylation studies use the traditional bisulfite treatment approach to measure 5-mC. Because bisulfite treatment alone cannot distinguish between 5-mC and 5-hmC^25^, some methylation signals may have been from 5-hmC. Further studies that explicitly distinguish between 5-hmC and 5-mC are needed to gain a clearer understanding of the effects of DNA cytosine modifications on gene expression.

We identified two genes, *HDAC4* and *IGF1R*, in our pediatric CNS tumors that were both epigenetically and transcriptionally altered in comparison to non-tumor pediatric brain tissue. *HDAC4* and *IGF1R* had differentially hydroxymethylated CpGs and increased expression in oligodendrocyte precursor cells across all four of our tumor types. Our results suggest a potential role of hydroxymethylation regulating genes associated with tumorigenesis. With these targets already having been studied in adult cancers, there are pharmacological inhibitors that already exist for these targets. Our study expands previously suggested ideas of targeting *HDAC4* and *IGF1R* in certain pediatric CNS tumor types^125,136,137^.

Accruing a large sample size for pediatric CNS tumors is extremely difficult as they are very rare in the general population. While our study does incorporate a decent sample size for these rare tumors, the smaller sample size limited the inclusion of other variables and cell types that may affect methylation and transcription into our models. Future studies with an expanded cohort of pediatric CNS patients will allow us to assess the epigenomic alterations in additional cell types of interest, such as glial cells. Moreover, following our findings of cell type-specific changes in DNA cytosine modifications in these pediatric CNS tumors, other tumor types may also have cell type-specific that have yet to be detected. Tools to understand the cell type composition of tissues should be incorporated in bulk epigenome-wide association studies to discriminate the cell type composition effects.

## Conclusion

Our study addresses gaps that currently exist in understanding epigenomic alterations at the cell type level in pediatric central nervous system tumors. Changes in hydroxymethylation were particularly drastic in progenitor-like cells and were associated with cell type level alterations in transcription. We highlight the relevance of epigenome dysregulation in pediatric central nervous system tumors that may lead us to more effective therapeutic targets.

## Methods

### Sample information

Cytosine modifications, bulk tissue gene expression, and single nuclei gene expression were measured in 32 pediatric CNS tumors of various types and 2 non-tumor pediatric brain tissue (**Table 1, Supplementary Table 1**). This study was approved by the Institutional Review Board Study #00030211. Only samples with all four molecular measurements were included in downstream analyses. The samples were collected from patients being treated at Dartmouth-Hitchcock Medical Center and the Dartmouth Cancer Center from 1993 to 2017. For each tumor type, the number of samples was distributed evenly with 8 samples for astrocytoma, 6 for embryonal tumors, 10 for ependymoma, and 8 for glioneuronal/neuronal tumors. Pathological re-review for the histopathologic tumor type and grade were done according to the 2021 World Health Organization CNS tumor classification system, then categorized into broader tumor types. The non-tumor pediatric brain tissues were obtained from patients who underwent surgical resection for epilepsy.

### Data collection and pre-processing

#### Single nuclei RNA-sequencing

The protocol to obtain single nuclei RNA-sequencing data and initial pre-processing steps were described in Chapter 3. To summarize briefly, nuclei were isolated from fresh frozen tissue samples following the Nuclei Pure Prep nuclei isolation kit (Sigma-Aldrich, St. Louis, MO). Each sample was multiplexed with lipid-tagged oligonucleotides following the MULTI-seq protocol^138^. Libraries for single nuclei RNA-seq were prepared following the 10X Genomics Single Cell Gene Expression workflows (10X Genomics, Pleasanton, CA). Libraries were pooled and sequenced using the lllumina NextSeq500 instrument. 10X Cell Ranger software was used to align sequences to the GRCh38 pre-mRNA reference genome.

Low-quality nuclei, as defined as having greater than 10,000 and less than 2,000 features and more than 5% of reads that map to mitochondrial genes, were removed for analyses. Samples were demultiplexed using an integrative approach, combining barcode based demultiplexing and genotype-based demultiplex method^139,140^. Downstream analyses for single nuclei-RNA seq were done with the Seurat package v4 in R^139,141–143^.

#### Bulk RNA-sequencing

Unused nuclei from our single nuclei RNA-seq experiment were used for bulk RNA-sequencing. RNA was isolated following the RNeasy Plus kit (Qiagen, Hilden, Germany). Libraries for bulk RNA-seq were prepared following the Takara Pico v3 low-input protocol (Takara Bio, Kusatsu, Japan).

Quality control for raw single-end RNA-seq data was checked using FastQC v0.11.8^144^. Reads were trimmed of polyA sequences and low-quality bases using Cutadapt v2.4^145^. Reads were aligned to the human pre-mRNA genome GRCh38 with STAR v2.7.7a^146^. Quality control of aligned reads was confirmed with *CollectRNASeqMetrics* in the Picard software v2.18.29^147^. Duplicate reads were identified with *MarkDuplicates* function in the Picard software^147^. One sample with an extremely high duplicate read percentage was removed from downstream analyses. Counts per gene were estimated using the *htseq-count* function in the HTseq software v0.11.2^148^.

#### DNA methylation and hydroxymethylation

In total, DNA from 33 paired pediatric brain tumor samples was treated with tandem bisulfite and oxidative bisulfite conversion followed by hybridization to Infinium HumanMethylationEPIC BeadChips to measure DNA methylation (5-mC) and hydroxymethylation (5-hmC). Raw BeadArray data were preprocessed using the *SeSAMe* pipeline from Bioconductor, including data normalization and quality control^149^. Cross-reactive probes, SNP-related probes, sex chromosome probes, non-CpG probes, and low-quality probes (pOOBHA > 0.05) were masked in the analysis^150^. The *oxBS.MLE* function was used to infer 5-mC and 5-hmC levels^151^.

#### Tumor purity estimates

Tumor purity for the tissue samples with DNA cytosine modifications was estimated using the *getPurity* function with the non-tumor pediatric tumor tissue as our non-tumor reference and the low-grade glioma (LGG) option as our cancer type in the InifiniumPurify package v1.3.1 in R^152^.

### Statistical analyses

#### Epigenome-wide association studies

Linear regression models, adjusting for sex, age at diagnosis, and tumor purity in all models, were used to identify differentially methylated and hydroxymethylated CpGs associated with each tumor type compared to the non-tumor tissue. Multiple linear regression models, with adjustments for different cell type proportions identified from the single nuclei RNA-seq data, were added to the models. Linear regression models were fit by using *lmFit* and *eBayes* functions in the limma package in R^153^. CpGs were considered differentially methylated or hydroxymethylated under the q-value threshold of 0.05.

Cell type-specific differential hydroxymethylation and methylation for each tumor type were identified using CellDMC^103^. Proportions of cell types of interest (neurons and progenitor-like cell types) were pulled from the single nuclei RNA-seq dataset. To limit overfitting the model in our relatively smaller sample size, we aggregated the progenitor-like cell types into a single cell type category. The progenitor-like cell types included neural stem cells (NSC), radial glial cells (RGC), oligodendrocyte precursor cells (OPC), and unipolar brush cells (UBC). UBCs were included due to the high levels of stemness score in the cell types identified previously.

#### Differential gene expression testing

Negative binomial regression models were used to identify the differential expressed genes in each tumor type compared to non-tumor tissue. One model was fit adjusting for age at diagnosis and sex. One model was fit adjusting for age at diagnosis, sex, and the proportions for cell types of interest (NEU, NSC, RGC, OPC, UBC), Negative binomial models were fit by using *DESeq* function in the DESeq2 package v1.36.0 in R^154^. Genes were considered as differentially expressed under the adjusted p-value threshold of 0.05.

#### Pathways enrichment testing

Reactome pathways enrichment associated with differentially expressed genes in each tumor type were identified using the *enrichPathway* function in the ReactomePA package v1.40.0 in R^155^.

#### Genomic context enrichment test

Enrichment tests for genomic context for differentially hydroxymethylated CpGs were conducted using the Mantel-Haenszel test. The MH test was adjusted for the type of probe (Type I or Type II) used for the CpG in the Illumina Methylation EPIC array.
